# Glucocorticoids Inhibit CRH/AVP-Evoked Bursting Activity of Male Murine Anterior Pituitary Corticotrophs

**DOI:** 10.1210/en.2016-1115

**Published:** 2016-06-02

**Authors:** Peter J. Duncan, Joël Tabak, Peter Ruth, Richard Bertram, Michael J. Shipston

**Affiliations:** Centre for Integrative Physiology (P.J.D., M.J.S.), College of Medicine and Veterinary Medicine, University of Edinburgh, Edinburgh EH8 9XD, United Kingdom; Biomedical Neuroscience Research Group (J.T.), University of Exeter Medical School, Exeter EX4 4PL, United Kingdom; Division of Pharmacology, Toxicology, and Clinical Pharmacy (P.R.), Institute for Pharmacy, University of Tübingen, D-72076 Tübingen, Germany; and Department of Mathematics and Programs in Neuroscience and Molecular Biophysics (R.B.), Florida State University, Tallahassee, Florida 32306

## Abstract

Corticotroph cells from the anterior pituitary are an integral component of the hypothalamic-pituitary-adrenal (HPA) axis, which governs the neuroendocrine response to stress. Corticotrophs are electrically excitable and fire spontaneous single-spike action potentials and also display secretagogue-induced bursting behavior. The HPA axis function is dependent on effective negative feedback in which elevated plasma glucocorticoids result in inhibition at the level of both the pituitary and the hypothalamus. In this study, we have used an electrophysiological approach coupled with mathematical modeling to investigate the regulation of spontaneous and CRH/arginine vasopressin-induced activity of corticotrophs by glucocorticoids. We reveal that pretreatment of corticotrophs with 100 nM corticosterone (CORT; 90 and 150 min) reduces spontaneous activity and prevents a transition from spiking to bursting after CRH/arginine vasopressin stimulation. In addition, previous studies have identified a role for large-conductance calcium- and voltage-activated potassium (BK) channels in the generation of secretagogue-induced bursting in corticotrophs. Using the dynamic clamp technique, we demonstrated that CRH-induced bursting can be switched to spiking by subtracting a fast BK current, whereas the addition of a fast BK current can induce bursting in CORT-treated cells. In addition, recordings from BK knockout mice (BK^−/−^) revealed that CORT can also inhibit excitability through BK-independent mechanisms to control spike frequency. Thus, we have established that glucocorticoids can modulate multiple properties of corticotroph electrical excitability through both BK-dependent and BK-independent mechanisms.

The ability of an organism to respond appropriately to stress is essential for survival when faced with a challenge to homeostasis. Stressful stimuli are associated with a rise in plasma glucocorticoids (cortisol in man, corticosterone in rodents, referred to as CORT), which are acutely beneficial, but chronic elevation can have many consequences on health (1–3). The neuroendocrine response to stress is governed by the hypothalamic-pituitary-adrenal (HPA) axis and is generally characterized by a surge of ACTH, which is subsequently switched off through elevation of plasma glucocorticoids that feed back directly on the pituitary or indirectly through higher centers of the brain (4). It is therefore important to understand the mechanisms of glucocorticoid-negative feedback at the cellular level, which is essential for the maintenance of effective HPA axis function.

The ability of glucocorticoids to suppress ACTH secretion has been well documented (5–7). Glucocorticoid-negative feedback can be divided into three distinct time domains: 1) rapid nongenomic, evident within seconds to minutes; 2) intermediate, early delayed, within 3 hours; and 3) slow, late delayed, which takes several hours to days (5). Rapid, nongenomic effects have been observed during in vivo rat studies in which the injection of CORT immediately prior to CRH resulted in a significant decrease in CRH-induced plasma ACTH levels compared with controls (8). Slow glucocorticoid-negative feedback is associated with a down-regulation of CRH receptors in corticotrophs (9) in addition to a decrease in ACTH expression (10, 11). The intermediate effects of glucocorticoid-negative feedback are likely to be mediated by a combination of genomic and nongenomic components.

Corticotroph cells of the anterior pituitary are a central component of the HPA axis integrating input from the hypothalamus while regulating CORT output from the adrenal cortex. Corticotrophs are electrically excitable and have been shown to fire single-spike action potentials (typical duration of depolarization phase <50 msec) as well as secretagogue-induced pseudoplateau bursting behavior, resulting in sustained depolarizations of more than 100 milliseconds' duration with small oscillations of the membrane potential during the depolarized phase of the burst, rather than full spikes (12, 13). In corticotrophs, this bursting is primarily controlled by activation of the CRH-signaling pathway, whereas the other major hypothalamic secretagogue, arginine vasopressin (AVP), promotes an increase in action potential frequency (13). Thus, the physiological response of corticotrophs after exposure to the combination of CRH and AVP, which would be released after a typical stressor, would be to promote both bursting and increase in single spike frequency (13). Bursting is an important feature of many anterior pituitary cell types (14) and raises intracellular Ca^2+^ to a greater extent than spiking and is proposed to underlie secretagogue-induced ACTH secretion (15–17).

Experimental data and mathematical modeling have identified that a large conductance Ca^2+^- and voltage-activated potassium (BK) channels are key regulators of bursting behavior in several different anterior pituitary cell types, including corticotrophs (13, 18, 19). Activation of a fast BK current (τ < 10 msec), during the upstroke of an action potential, prevents the activation of delayed rectifier K^+^ channels, which holds the cell in a depolarized state longer than would be the case without BK channels, producing so-called pseudoplateau bursting (14). BK channels have been reported to be regulated by glucocorticoids, in a number of different systems, by a variety of mechanisms. This includes direct effects on BK channel activity, mediated via accessory subunits (20, 21); regulation of BK channels through protein dephosphorylation (22, 23), and switching of BK channel splice variant expression (24, 25). However, whether glucocorticoids control the electrical excitability of native corticotrophs and/or whether this is through control of BK channel function are not known.

In this study, we used an electrophysiological approach coupled with mathematical modeling to interrogate the regulation of corticotroph excitability by glucocorticoids. We reveal that CORT is able to modulate both spontaneous and secretagogue-evoked excitability of corticotrophs. In the intermediate time domain, CORT suppresses CRH-evoked bursting behavior through a BK channel dependent mechanism. Using dynamic clamp, subtraction of an artificial BK conductance can suppress CRH-induced bursting activity. Conversely, the addition of a fast BK current can restore CRH-evoked bursting in CORT-treated cells. In addition, CORT reduces both spontaneous and evoked firing frequency in a mechanism independent of BK channels. Thus, glucocorticoids differentially regulate corticotroph excitability over multiple time domains through both BK-dependent and BK-independent mechanisms.

## Materials and Methods

### Reagents

General biochemical reagents used throughout this study were obtained from Sigma-Aldrich and were of analytical-grade quality unless stated otherwise.

### Animals

Mice lacking the pore-forming exon of the BK channel α-subunit (26) were crossed with mice expressing green fluorescent protein (GFP) under the proopiomelanocortin (POMC) promoter (27) to generate BK-POMC-GFP mice with a SV129/C57BL6 mixed background and crossed for at least 10 generations. Mice were caged in groups of two to four under standard laboratory conditions (lights on at 7:00 am, lights off at 7:00 pm, 21°C, with tap water and chow available ad libitum) at the University of Edinburgh. Wild-type, or mice deficient for the BK channel (BK^−/−^), were used from the same litters generated by a cross of mice heterozygous for the BK allele. Tissue collection was performed between 8:30 am and 10:00 am in accordance with United Kingdom Home Office requirements (PPL 60/4349) and University of Edinburgh Ethical Review Committee approval.

### Pituitary cell culture

Corticotroph cells were acutely isolated by trypsin digestion from male mice (aged 2–5 mo) constitutively expressing GFP under the control of the POMC promoter (POMC-GFP) as previously described (13). Male corticotrophs were used in these studies as in female corticotrophs from randomly cycling animals the response to CRH/AVP is more variable, the mean duration of individual evoked pseudoplateau bursts is shorter, and they express intermediate conductance calcium-activated potassium channels that also contribute to bursting (12, 13). Cells were cultured on 12-mm coverslips in serum-free media (DMEM containing 25 mM HEPES, 5 μg/mL insulin, 50 μg/mL transferrin, 30 nM sodium selenite, 0.3% BSA [wt/vol], 4.2 μg/mL fibronectin, and antibiotic/antimycotic [100× dilution of Sigma stock]) and incubated at 37°C in 5% CO_2_. Serum-free media (lacking antibiotic/antimycotic) was changed every 2 days and electrophysiological recordings were obtained from cells 24–96 hours after isolation.

### Electrophysiology

Electrophysiological recordings were obtained from corticotrophs using the perforated patch mode of the whole-cell patch clamp technique. Amphotericin B was used at a concentration of 150 μg/mL in pipette solution, which resulted in access resistances typically less than 40 MΩ within 10–20 minutes and allowed stable recordings in excess of 40 minutes. The standard bath (extracellular) solution contained the following (in millimoles): 140 NaCl, 5 KCl, 2 CaCl_2_, 0.1 MgCl_2_, 10 HEPES, and 10 glucose. The pH was adjusted to 7.4 with NaOH, 300 mOsmol/L. The standard pipette (intracellular) solution contained the following (in millimoles): 10 NaCl, 30 KCl, 60 K_2_SO_4_, 1 MgCl_2_, 10 HEPES, 10 glucose, and 50 sucrose. The pH was adjusted to 7.2 with KOH, 290 mOsmol/L.

Electrophysiological recordings were performed at room temperature (18°C–22°C) to facilitate stable recordings of more than 30 minutes required for these assays and obtained using Clampex 10.1 (Molecular Devices) with a sampling rate of 10 kHz and filtered at 2 kHz. Patch pipettes were fabricated from borosilicate glass (King Precision Glass, Inc) using a model P-97 micropipette puller (Sutter Instruments). Pipette tips were heat polished and had resistances typically between 2 and 3 MΩ. Compensated series resistance was typically less than 20 MΩ and capacitance of corticotrophs ranged from 2 to 10 pF. A gravity-driven perfusion system was used to apply drugs to the cells with a flow rate of 1–2 mL/min to minimize flow-induced artifacts.

### CRH and/or AVP stimulation and glucocorticoid pretreatment

Initial studies were performed using a 3-minute pulse of combined CRH and AVP (0.2 and 2 nM, respectively; Bachem), which represent physiologically relevant concentrations found in the portal circulation in response to stress (28, 29). To interrogate the role of BK channels in bursting per se, which is driven by CRH but not AVP (13), CRH alone (3 min, 0.2 nM) was used as secretagogue in dynamic clamp assays.

To analyze the role of glucocorticoid regulation of excitability in different time domains, 100 nM free CORT was used for pretreatment in vitro. This represents free CORT levels typically observed in plasma in vivo, after an acute stress in rodents (30). Rapid actions of CORT were investigated by pretreating cells with 100 nM CORT in electrophysiological bath solution for 4 minutes (CORT_4_) prior to CRH/AVP exposure. To investigate longer time domains of glucocorticoid-negative feedback, corticotrophs were first pretreated with 100 nM CORT in culture media containing 0.3% BSA at 37°C for either 60 or 120 minutes, conditions in which the free CORT is estimated at approximately 70 nM (31). At the end of the pretreatment phase, corticotrophs were transferred to electrophysiological bath solution (without BSA) containing 100 nM CORT during cell identification and patch perforation (∼30 min). Thus, total pretreatment times analyzed were 90 minutes (CORT_90_) and 150 minutes (CORT_150_) unless indicated otherwise. In all pretreatment groups, 100 nM CORT was present during CRH/AVP exposure and remained during the CRH/AVP washout period.

### Dynamic clamp

A separate digital acquisition card and computer were used to run a dynamic clamp module within the software QuB (32). In the current clamp mode of the patch amplifier (Axopatch 200B; Molecular Devices), membrane potential (*V*) was used to compute the current going through the BK channels, *I_BK_* = *g_BK_f*(*V_K_* − *V*), with *f* obtained by integrating

(1) $$\tau_{BK}\frac{df}{dt}-f_{\infty }\left(V\right)-f$$

in real time using the forward Euler method (32), with an average time step of 21 μsec (maximum ≤100 μsec), and the steady-state BK channel activation given by

(2) $$f_{\infty }\left(V\right)={\left[1+\exp\left(\frac{v_{f}-V}{s_{f}}\right)\right]}^{-1}$$

The calculated BK current was injected back into the cell through the same digital acquisition card. Usual parameter values were as follows: *g_BK_* = 0.5 nS; τ*_BK_* = 5 msec; *v_f_* = −10 mV; *s_f_* = 2 mV.

### Electrophysiology analysis

Current clamp recordings were analyzed as previously described (13) using Clampfit version 10.1 (Molecular Devices). Secretagogue-evoked activity was measured immediately after 3 minutes of CRH/AVP stimulation. In addition to the mean event duration, bursting behavior was quantified through the calculation of a burstiness factor. This method classifies any event less than 100 milliseconds as a spike and events greater than 100 milliseconds as a burst; a burstiness factor is calculated as the fraction of events that are bursts (13, 19).

The data were expressed as mean ± SEM. The number of independent experiments per group was 8 throughout, except for dynamic clamp studies in which the number was six per group. Statistical analysis was performed as appropriate by a Student's *t* test (Microsoft Excel) or a two-way ANOVA with Bonferroni post hoc multiple comparison tests (GraphPad Prism 6). Significant differences between groups were defined (*, *P* < .05 and **, *P* < .01).

### Mathematical model

We use a biophysical model to simulate the effects of BK channel knockout and application of CRH, AVP, and CORT. We used this model in a previous publication (13), in which it was described in some detail, so we give an abbreviated description here. The model consists of six ionic currents: L-type Ca^2+^ current (*I_Ca_*), delayed rectifier K^+^ current (*I*_*K* − *dr*_), inward rectifier K^+^ current (*I*_*K* − *ir*_), BK-near (*I*_*BK* − *near*_) and BK-far (*I*_*BK* − *far*_) currents, and a nonselective current (*I_NS_*). Parameter values are given in Table 1, and the computer programs used to generate the modeling figures can be downloaded (http://www.math.fsu.edu/∼bertram/software/pituitary). The model differential equations were solved numerically using the XPPAUT software program (33) using an Euler method with step size dt = 0.01 milliseconds.

**Table 1.** Default Parameter Values for the Corticotroph Model

Parameter	Value	Parameter	Value
*g_Ca_*	1.8 nS	*k*_*CaBK*−*near*_	2 μM
*g_NS_*	0.12 nS	*k*_*CaBK*−*far*_	6 μM
*g_K−dr_*	8.2 nS	*k_BK_*	3 mV
*g_K−ir_*	1 nS	*sm*	10 mV
*g_BK−near_*	2 nS	*sn*	10 mV
*g_BK−far_*	1 nS	*sr*	−1 mV
*V_Ca_*	60 mV	*V_BK0_*	0.1 mV
*V_NS_*	−10 mV	*k_shift_*	18 mV
*V_K_*	−75 mV	*A*	0.12 μM msec fC^−1^
*V_r_*	−60 mV	*k_C_*	0.12 msec^−1^
*V_m_*	−20 mV	*f*	0.01
*V_n_*	−5 mV	α	0.0015 μMfC^−1^
τ*_bkn_*	20 msec	σ*_N_*	5 pA
τ*_bkf_*	3 msec	*C_m_*	6 pF
τ*_n_*	40 msec		

The cell's membrane potential (*V*) is described by the following equation:

(3) $$C_{m}\frac{dV}{dt}=-\left(I_{Ca}-I_{K-dr}+I_{BK-near}+I_{BK-far}+I_{K-ir}+I_{NS}+I_{noise}\right)$$

where *C_m_* is the membrane capacitance and the first six terms on the right-hand side are ionic currents. The last term reflects channel noise. It is given by *I_noise_* = σ*_N_*ω where σ*_N_* is the noise amplitude and ω is a random Wiener variable. The ionic currents are as follows:

(4) $$\text{L}-\text{type}\;{\text{Ca}}^{2+}\;\text{current}:\;I_{Ca}\left(V\right)=g_{Ca}m_{\infty }\left(V\right)\left(V-V_{Ca}\right)$$

(5) $$\text{Delayed}\;\text{rectifying}\;{\text{K}}^{+}\;\text{current}:\;I_{K-dr}\left(V\right)=g_{K-dr}n\left(V-V_{K}\right)$$

(6) $$\text{Inward}\;\text{rectifying}\;{\text{K}}^{+}\;\text{current}:\;I_{K-ir}\left(V\right)=g_{K-ir}r_{\infty }\left(V\right)\left(V-V_{K}\right)$$

(7) $$\text{Non}-\text{Selective}\;\text{current}:\;I_{NS}\left(V\right)=g_{NS}\left(V-V_{NS}\right)$$

(8) $$\text{BK}-\text{far}\;{\text{K}}^{+}\;\text{current}:\;I_{BK-far}\left(V,c\right)=g_{BK-far}bk_{f}\left(V-V_{K}\right)$$

(9) $$\text{BK}-\text{near}\;{\text{K}}^{+}\;\text{current}:\;I_{BK-near}\left(V,c_{dom}\right)=g_{BK-near}bk_{f}\left(V-V_{K}\right)$$

The nonselective current has constant conductance and is generally depolarizing. The spikes are produced by the interaction of the Ca^2+^ current and the delayed rectifier, and for simplicity the Ca^2+^ current is assumed to activate instantaneously, as does the inward rectifier. There are two populations of BK channels in the model. The BK-near channels are located close to Ca^2+^ channels and are activated by Ca^2+^ microdomains that form at the inner mouth of an open Ca^2+^ channel. The BK-far channels are located further from the Ca^2+^ channels and respond to the bulk cytosolic Ca^2+^, which has a much lower concentration. The domain Ca^2+^ is given by *c_DOM_* = −*AI_Ca_*(*V*), where *A* is a parameter that converts Ca^2+^ current to Ca^2+^ concentration. The bulk cytosolic Ca^2+^ concentration, *c*, changes with time according to the following:

(10) $$\frac{dc}{dt}=-f\left(\alpha I_{Ca}+k_{c}C\right)$$

where *f* is the fraction of Ca^2+^ that is free, α converts current to concentration change, and *k_c_* is the pump rate of plasma membrane Ca^2+^ ATPases.

The activation variables all satisfy first-order kinetics, with *V*-dependent steady-state functions of the Boltzmann form:

(11) $$x_{\infty }\left(V\right)=\frac{1}{1+e\left(\frac{v_{x}-V}{s_{x}}\right)},\;x=n,m,r$$

and the dynamics for gating variables that do not activate instantaneously are described by the following equations:

(12) $$\frac{dn}{dt}=\frac{n_{\infty }\left(V\right)-n}{\tau_{n}}$$

(13) $$\frac{dbk_{n}}{dt}=\frac{bk_{n\infty }\left(V,c_{DOM}\right)-bk_{n}}{\tau_{bk_{n}}}$$

(14) $$\frac{dbk_{f}}{dt}=\frac{bk_{f\infty }\left(V,c\right)-bk_{f}}{\tau_{bk_{f}}}$$

with the equilibrium functions

(15) $$bk_{n\infty }\left(V,c_{DOM}\right)=\frac{1}{1+\exp\frac{-\left(V-V_{BK-near}\left(c_{DOM}\right)\right)}{k_{BK}}}$$

(16) $$bk_{f\infty }\left(V,c\right)=\frac{1}{1+\exp\frac{-\left(V-V_{BK-far}\left(c\right)\right)}{k_{BK}}}$$

where the half-activation voltage shifts with the domain or bulk cytosolic Ca^2+^ concentration according to the following equations:

(17) $$V_{BK-near}\left(c_{DOM}\right)=V_{BK_{0}}-k_{shift}\;\text{In}\frac{c_{DOM}}{k_{Ca_{BK-near}}}$$

(18) $$V_{BK-far}\left(c\right)=V_{BK_{0}}-k_{shift}\;\text{In}\frac{c}{k_{Ca_{BK-far}}}.$$

In the model, under basal conditions, the BK-near population have an activation curve that is significantly left shifted compared with that of BK-far (*V*_*BK*−*near*_ < *V*_*BK*−*far*_) and with a slower activation time constant compared with BK-far (τ*_bk_n__* > τ*_bk_f__*). After stimulation with CRH, the activation curve becomes right shifted (toward that of BK-far) and the activation time constant becomes the same as BK-far (13).

## Results

### CORT reduces spontaneous and CRH/AVP-evoked corticotroph electrical excitability

**Figure 1.**
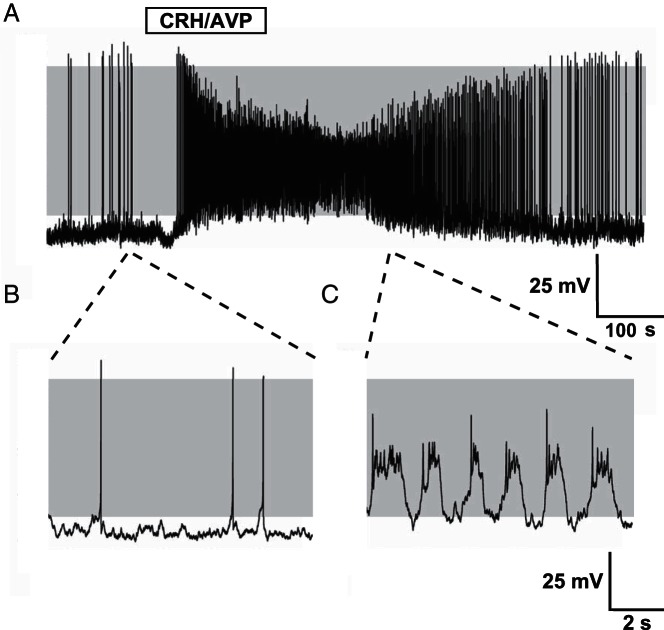
Stimulation of corticotrophs with CRH/AVP. Representative current clamp recording of a corticotroph cell exposed to 0.2 nM CRH and 2 nM AVP for 3 minutes (A). Corticotrophs display predominantly single-spike action potentials at rest (B) and CRH/AVP stimulation results in an increase in frequency and a transition to bursting behavior (C). Gray shading indicates membrane potential between −50 and +10 mV.

**Figure 2.**
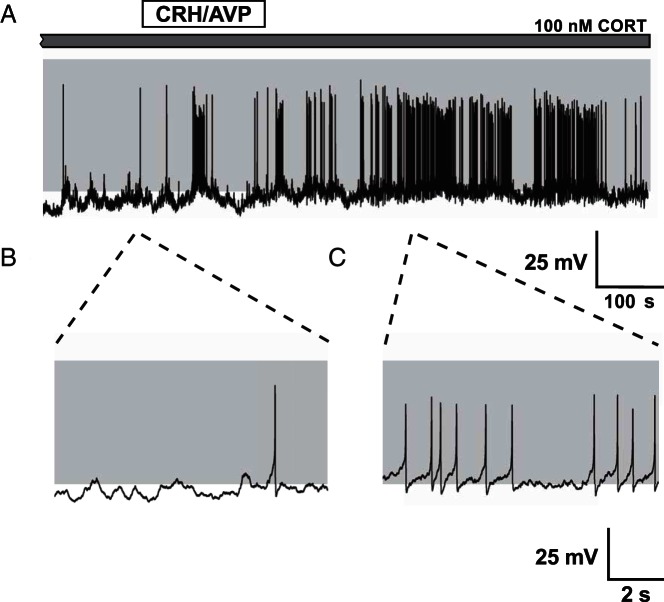
CRH/AVP stimulation of CORT-treated cells. Corticotrophs pretreated with CORT (100 nM) for 90 minutes show a reduction in spontaneous and CRH/AVP-evoked activity (A). CORT treatment results in a membrane hyperpolarization and a reduction in spontaneous firing frequency (B). CRH/AVP is still able to induce an increase in activity but fails to cause a significant transition from spiking to bursting (C).

Spontaneous activity was observed in the majority (>90%) of untreated cells and consisted predominantly of large amplitude, single-spike action potentials. Occasionally spontaneous bursting events (>100 msec duration) were observed alongside single-spike action potentials but these events were rare (<6% of all corticotrophs analyzed). Stimulation of corticotrophs with physiological concentrations of CRH/AVP (0.2 and 2 nM, respectively) resulted in a robust depolarization and an increase in event frequency (Figure 1A) coupled with a transition from spiking (Figure 1B) to bursting (Figure 1C). To investigate the different time domains of glucocorticoid-negative feedback, corticotrophs were pretreated with 100 nM CORT for 4 minutes (CORT_4_), 90 minutes (CORT_90_), or 150 minutes (CORT_150_) prior to CRH/AVP exposure (n = 8/group throughout). Current clamp recordings from CORT_90_ cells (Figure 2A) revealed a decrease in spontaneous activity (Figure 2B) and a failure to transition to bursting after CRH/AVP stimulation (Figure 2C).

**Figure 3.**
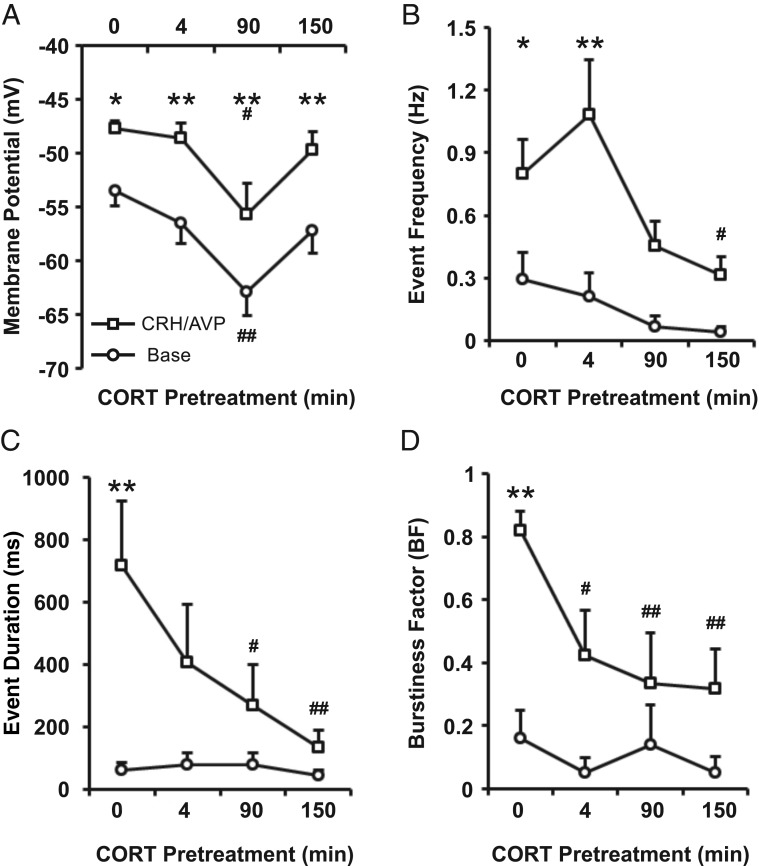
CORT regulates basal and secretagogue-induced activity of corticotrophs. Corticotrophs were treated with CORT (100 nM) for 4, 90, and 150 minutes prior to CRH/AVP stimulation. CORT pretreatment results in a membrane hyperpolarization (A) and a decrease in both spontaneous and CRH/AVP-evoked event frequency (B). Additionally, CORT inhibits CRH/AVP-induced bursting activity represented by a decrease in mean event frequency (C) and burstiness factor (D). Data are means ± SEM (n = 8 per group). *, *P* < .05, **, *P* < .01 compared with respective base values; #, *P* < .05, ##, *P* < .01 compared with control (untreated cells) (two way ANOVA with Bonferroni post hoc analysis).

Resting membrane potential of control cells (n = 8) was −53.5 ± 1.4 mV and 3 minutes of CRH/AVP stimulation resulted in a significant (*P* < .05) membrane depolarization to −47.7 ± 0.73 mV (Figure 3A), which returned to −52.5 ± 1.1 mV by the end of the recording period and was not significantly different from baseline. Acute CORT treatment (CORT_4_ cells) had no significant effect on resting membrane potential or CRH/AVP-evoked depolarization compared with controls (n = 8). Cells treated with CORT for 90 minutes (98 ± 3 min; n = 8) were significantly (*P* < .01) hyperpolarized (−62.9 ± 2.2 mV) at rest compared with control cells. CRH/AVP induced a significant (*P* < .01) depolarization in CORT_90_ cells, although membrane potential remained significantly (*P* < .05) hyperpolarized (−55.7 ± 2.9 mV) compared with CRH/AVP-evoked membrane potential in control cells (Figure 3A). Interestingly, this hyperpolarization was absent in corticotrophs treated for 150 minutes (155 ± 3 min; n = 8), suggesting there are multiple phases to glucocorticoid regulation of resting membrane potential. CRH/AVP-evoked depolarization (δ 5.8 ± 1.6 mV) was not significantly different between control and CORT-treated groups, suggesting the mechanism for depolarization is not a target for CORT in the time domains investigated.

CRH/AVP-evoked membrane depolarization was accompanied by an increase in firing frequency. Control cells had a basal firing frequency of 0.29 ± 0.13 Hz, which significantly (*P* < .05) increased to 0.80 ± 0.17 Hz after CRH/AVP stimulation. Pretreatment with CORT inhibited CRH/AVP-induced electrical excitability in all groups; however, the effects of CORT were dependent on the time of CORT pretreatment. CRH/AVP increased event frequency significantly (*P* < .01) in CORT_4_ cells to 1.09 ± 0.26 Hz, similar to that observed with CRH/AVP in untreated cells. However, after the CRH/AVP washout period, event frequency decreased back to 0.32 ± 0.09 Hz in CORT_4_ cells, in contrast to untreated cells in which it remained elevated after 10 minutes after CRH/AVP washout (0.81 ± 0.22 Hz), suggesting a relatively rapid component to glucocorticoid feedback.

In contrast, CRH/AVP failed to produce a significant increase in event frequency in CORT_90_ and CORT_150_ cells (Figure 3B). CRH/AVP-evoked frequency was 0.31 ± 0.09 Hz in CORT_150_ cells, which was significantly (*P* < .05) reduced compared with the effect of CRH/AVP in control cells. The response of corticotrophs to CRH/AVP is rapid, with untreated cells responding with depolarization and onset of bursting within 32 ± 2 seconds after initial CRH/AVP exposure. The response delay increased as the time of CORT pretreatment increases and becomes significantly (*P* < .01) different from controls in CORT_150_ cells in which the delay is increased to 163 ± 50 seconds.

CRH/AVP-induced transition to bursting in corticotrophs was attenuated by CORT pretreatment (Figure 3, C and D). Under basal conditions, the mean event duration and burstiness factor of untreated cells were 63 ± 23 milliseconds and 0.16 ± 0.09 milliseconds, respectively, corresponding to single-spike activity. After the CRH/AVP stimulation, the mean event duration increased significantly (*P* < .01) to 718 ± 206 milliseconds (Figure 3C), and the burstiness factor increased significantly (*P* < .01) to 0.82 ± 0.06 (Figure 3D), representative of a transition to bursting behavior.

Under basal conditions, CORT pretreatment had no significant effect on either mean event duration or burstiness factor, which was not surprising because bursting events were uncommon in corticotroph cells at rest. CRH/AVP failed to significantly increase event duration in CORT_90_ cells, which have a CRH/AVP-evoked event duration of 270 ± 130 milliseconds. The effect is enhanced in CORT_150_ cells (133 ± 56 msec), which is significantly (*P* < .01) reduced compared with controls (Figure 3C). After the CRH/AVP stimulation, the burstiness factors of CORT_90_ and CORT_150_ cells were 0.33 ± 0.16 and 0.32 ± 0.13, respectively, and were significantly (*P* < .01) reduced compared with untreated cells (Figure 3D).

### CORT inhibits BK-dependent bursting as well as the BK-independent increase in spike frequency

Because the current data revealed that CORT largely prevents CRH/AVP-induced bursting in corticotrophs and bursting is dependent on functional BK channels in murine corticotrophs (13), we hypothesized that BK channels may be a key target for glucocorticoid-negative feedback in the intermediate time domain. We thus compared the effect of CORT on basal and CRH/AVP-evoked excitability between wild-type and BK^−/−^ corticotrophs (n = 8/group throughout).

**Figure 4.**
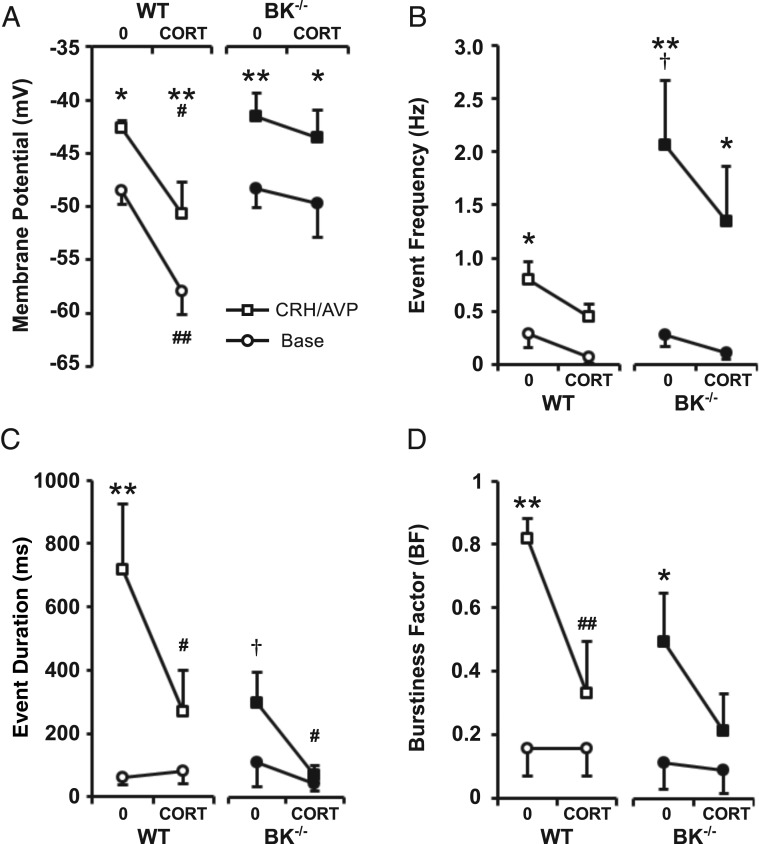
CORT regulates excitability independently of BK channels. Wild-type (WT) and BK^−/−^ cells were treated for 100 minutes with 100 nM CORT and compared with respective untreated controls (0). CRH/AVP stimulation evoked a significant depolarization in all groups (A). BK^−/−^ cells had an increased CRH/AVP-evoked frequency compared with wild types, and CORT can modulate frequency through BK-independent mechanisms (B). Secretagogue-induced bursting was decreased in BK^−/−^ cells, and CORT treatment resulted in a further decrease in mean event duration (C) and burstiness factor (D). Data are means ± SEM (n = 8 per group). *, *P* < .05, **, *P* < .01 compared with base values; #, *P* < .05, ##, *P* < .01 compared with respective untreated controls; †, *P* < .05 compared with untreated wild-type cells (two way ANOVA with Bonferroni post hoc analysis).

As previously described (13), corticotrophs isolated from BK^−/−^ mice (n = 8) showed no difference in basal excitability compared with wild-type cells. CRH/AVP was still able to evoke a significant (*P* < .01) membrane depolarization and increase in event frequency in BK^−/−^ cells (Figure 4, A and B). However, CRH/AVP-induced frequency was significantly (*P* < .05) higher in BK^−/−^ cells (2.07 ± 0.60 Hz) compared with wild types (0.80 ± 0.17 Hz). Genetic deletion of BK channels resulted in a reduction in secretagogue-induced bursting activity (Figure 4, C and D). Although CRH/AVP was able to induce a significant (*P* < .05) increase in burstiness factor in BK^−/−^ cells (from 0.12 ± 0.08 to 0.50 ± 0.15), event duration (295 ± 96 msec) was significantly (*P* < .05) reduced compared with CRH/AVP-stimulated wild-type cells (718 ± 206 msec).

Corticotrophs isolated from BK^−/−^ mice were treated with 100 nM CORT for 102 ± 4 minutes (n = 8) before exposure to CRH/AVP following the same protocol. CORT treatment of BK^−/−^ cells had no effect on resting membrane potential in contrast to the effect of CORT in wild-type cells (Figure 4A). However, as observed in wild-type cells, CRH/AVP was still able to induce a significant (*P* < .05) depolarization in CORT-treated BK^−/−^ cells, similar to that observed in untreated BK^−/−^ cells. Furthermore, the response delay for BK^−/−^ cells and CORT-treated BK^−/−^ cells was 57 ± 8 and 42 ± 15 seconds, respectively, and was not significantly different compared with untreated wild-type cells. This suggests that neither BK channels nor CORT feedback is a major determinant of the initial depolarization evoked by CRH/AVP.

CORT treatment of BK^−/−^ cells resulted in a reduction in both CRH/AVP-induced event frequency and bursting behavior compared with untreated BK^−/−^ cells. After CRH/AVP, stimulated event frequency was reduced in CORT-treated BK^−/−^ cells (1.35 ± 0.51 Hz) but was not significantly different from untreated BK^−/−^ cells (Figure 4B). CRH/AVP was unable to induce a significant increase in event duration in CORT-treated BK^−/−^ cells (67 ± 30 msec), which was significantly (*P* < .05) reduced compared with untreated BK^−/−^ cells (Figure 4C). Similarly, CRH/AVP was unable to increase burstiness factor in CORT-treated BK^−/−^ cells (0.22 ± 0.14) compared with prestimulation levels (Figure 4D). Taken together, these data suggest that BK channels promote the transition to bursting evoked by CRH/AVP but that some residual bursting can be maintained in the complete absence of BK channels as previously reported (13). However, the primary effect of CORT is to inhibit CRH/AVP-evoked BK channel-dependent bursting behavior.

### Modeling the effects of glucocorticoids on corticotroph excitability

Previous mathematical modeling of corticotroph excitability (13) predicted that CRH promotes bursting by increasing L-type Ca^2+^ conductance and regulating BK-near so that the BK-near voltage curve becomes right shifted, whereas it activates sufficiently rapidly (< 10 msec) in the presence of high Ca^2+^ next to L-type Ca^2+^ channels, whereas AVP increases single spike frequency by increasing a nonselective Na^+^ current. We therefore used the model to predict the underlying conductances involved in glucocorticoid-negative feedback.

**Figure 5.**
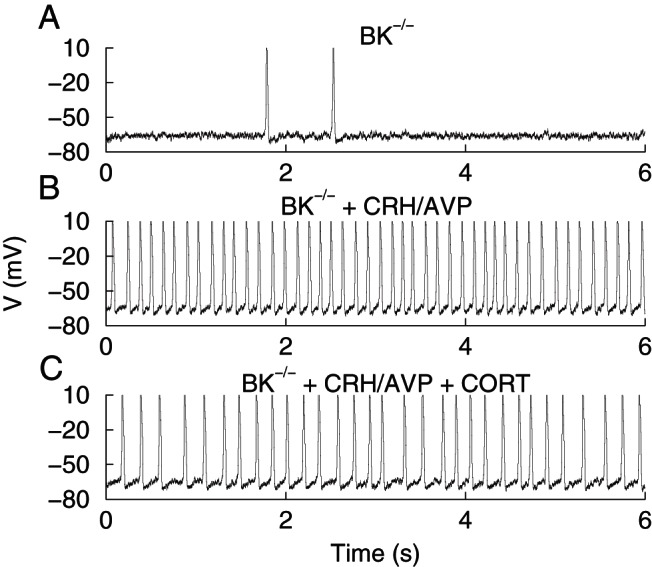
Simulations of model corticotrophs with BK conductance removed (*g*_*BK*−*near*_ = *g*_*BK*−*far*_ = 0). Basal activity exhibiting random spiking at a low rate (A). Application of CRH and AVP is simulated, greatly increasing the spike frequency with no bursting (B). CRH is simulated by increasing the Ca^2+^ current conductance (*g_Ca_* = 2.4 nS). Application of AVP is simulated by increasing the nonselective current conductance (*g_NS_* = 0.2 nS). In the presence of CORT, the spiking induced by CRH/AVP is slower (C). CORT is simulated by reducing the nonselective current conductance from its elevated AVP-induced value (*g_NS_* = 0.15 nS).

The experimental data showed that CRH/AVP-evoked firing frequency was higher in BK^−/−^ cells compared with wild-type cells but that bursting behavior was suppressed. CORT treatment of BK^−/−^ cells resulted in a reduction in CRH/AVP-induced spike frequency. BK^−/−^ cells were modeled by setting both BK-near and BK-far conductances to zero, and the unstimulated model cell displayed low frequency action potentials (Figure 5A). In the absence of BK channels, the effect of CRH was modeled by increasing the L-type Ca^2+^ conductance (*g_Ca_* = 2.4 nS), whereas AVP was simulated by increasing the nonselective Na^+^ conductance (*g_NS_* = 0.2 nS). This resulted in an increase in spike frequency without inducing bursting behavior (Figure 5B). The model shows that CRH/AVP-induced spike frequency may be reduced in CORT-treated BK^−/−^ cells by partially opposing the increase in the nonselective current conductance (*g_NS_* = 0.15 nS), which is mediated by AVP (Figure 5C).

**Figure 6.**
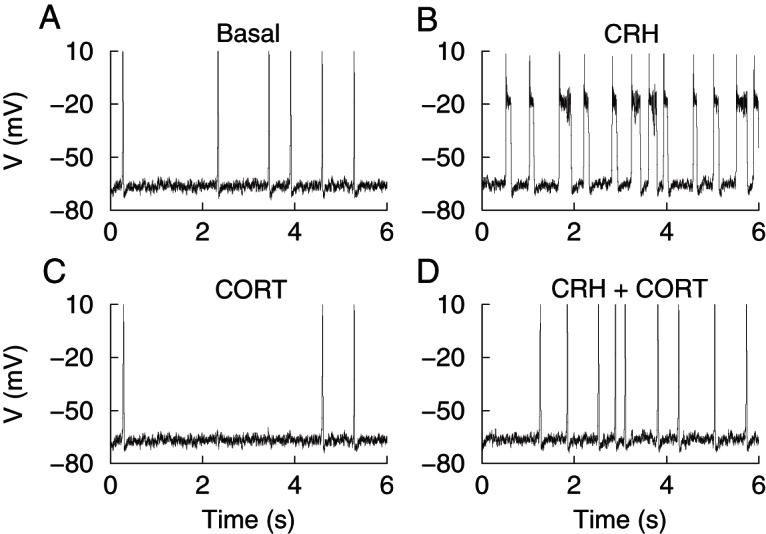
Simulations of the effects of CRH and CORT on model corticotrophs. In basal conditions the model cells spike at a low rate (A). Application of CRH increases the rate of activity, and bursting is prevalent (B). CRH is simulated by increasing the Ca^2+^ current conductance (*g_Ca_* = 2.4 nS), shifting the BK-near activation curve rightward by matching the Ca^2+^ affinity of the BK-far channels (*k*_*Ca*_*BK*−*near*__ = 6 μM), and the time constant for BK-near channel activation is decreased to match that of BK-far channel activation (τ*_BK_n__* = 3 msec). In the presence of CORT, the model cell spikes at a lower rate than under basal conditions (C). CORT is simulated by reducing the nonselective current conductance (*g_Ca_* = 0.11 nS). In the presence of CORT, CRH does not induce bursting in the model corticotroph, but it does increase the spike frequency (D). CRH in the presence of CORT is simulated by increasing the Ca^2+^ current conductance (*g_Ca_* = 2.4 nS) but with no direct effect on BK channels.

To examine the effects of CORT on bursting, we modeled the effect of CRH stimulation alone on wild-type cells as CRH, but not AVP, promotes bursting. In the absence of CORT, unstimulated corticotrophs display predominantly single-spike action potentials (Figure 6A). CRH mediates a transition to bursting by increasing L-type Ca^2+^ conductance (*g_Ca_* = 2.4 nS) while right shifting the BK-near activation curve by matching the Ca^2+^ affinity of the BK-far channels (*k*_*Ca*_*BK*−*near*__ = 6 μM) and decreasing the time constant for BK-near channel activation (τ*_BK_n__* = 3 msec) to match that of BK-far channel activation (Figure 6B).

Spontaneous single-spike activity is decreased in CORT-treated cells due to a reduction in the nonselective current (*g_NS_* = 0.11 nS), causing a modest hyperpolarization, which decreases the likelihood of the cell reaching threshold (Figure 6C). In the presence of CORT, CRH causes an increase in spike frequency but fails to mediate a transition to bursting (Figure 6D). The effects of CORT are simulated by preventing the modulation of BK-near channels by CRH, whereas the increase in L-type Ca^2+^ conductance is unaffected (*g_Ca_* = 2.4 nS).

### Fast-activating BK current is a crucial component of bursting behavior

To test experimentally whether fast-activating BK channels are an important component of CRH-induced bursting and a likely target for CORT inhibition of bursting, we used a dynamic clamp to ask whether the subtraction of a fast-activating BK conductance reversed CRH-induced bursting in control cells. Conversely, we tested whether the addition of a fast-activating BK current in CORT-treated cells could restore CRH-evoked bursting.

**Figure 7.**
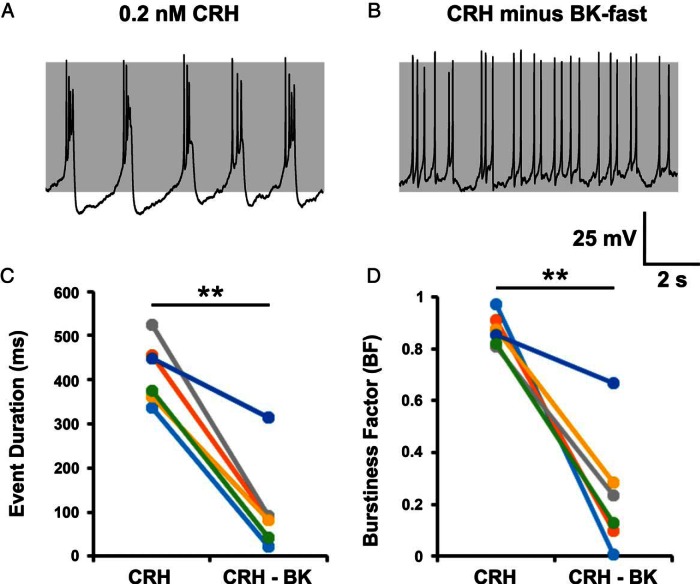
Subtraction of a fast BK current reduces CRH-induced bursting activity. Bursting behavior in CRH stimulated cells (A) could be switched to spiking by subtracting a fast BK current using dynamic clamp (B) and resulted in a significant decrease in both mean event duration (C) and burstiness factor (D) in all cells tested. In most cases, the parameters were as follows: *g_BK_* = −0.5 nS; *v_f_* = −10 mV; τ*_BK_* = 5 milliseconds. Data are shown for individual cells (n = 6 per group). **, *P* < .01 comparison of means (paired Student's *t* test).

Current clamp recordings were obtained from control cells stimulated for 3 minutes with CRH alone (0.2 nM, n = 6). After CRH stimulation, bursting was observed in all cells (Figure 7A), resulting in a CRH-induced event duration of 417 ± 29 milliseconds and a burstiness factor of 0.87 ± 0.03 (Figure 7, C and D). Subtraction of a fast BK current, with a dynamic clamp in CRH-stimulated cells (Figure 7B) caused a significant (*P* < .01) reductions in both event duration (107 ± 43 msec) and burstiness factor (0.24 ± 0.10), resulting in a switch back to spiking or significant reduction in bursting activity. In most cases, the parameters were as follows: *g_BK_* = −0.5 nS; *v_f_* = −10 mV; τ*_BK_* = 5 milliseconds. However, due to the intrinsic variability of corticotroph activity, parameters were modified slightly from cell to cell.

**Figure 8.**
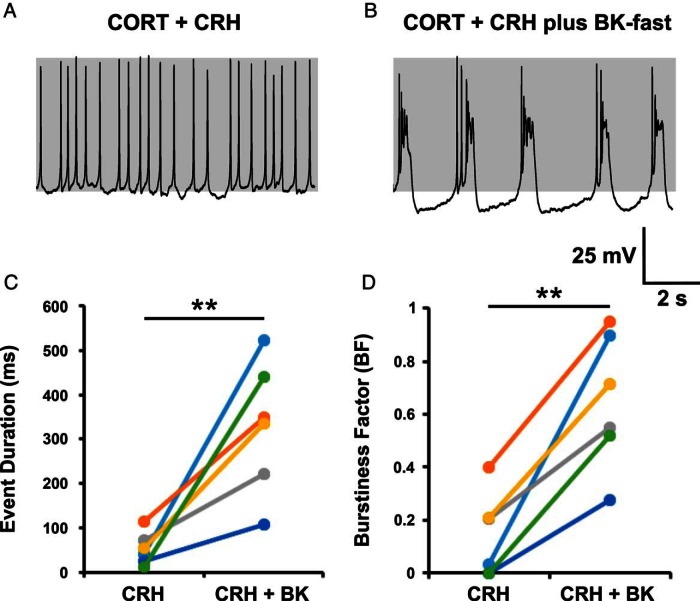
Addition of a fast BK current can induce bursting in CORT-treated cells. Corticotrophs treated with CORT (100 nM) for 150 minutes failed to transition to bursting after CRH stimulation (A). Bursting could be induced by adding a fast BK current with dynamic clamp (B) and resulted in a significant increase in mean event duration (C) and burstiness factor (D) in all cells tested. In most cases, the parameters were as follows: *g_BK_* = +0.5 nS; *V_f_* = −10 mV; τ*_BK_* = 5 milliseconds; *g_lk_* = 0.04 pS. Data are shown for individual cells (n = 6 per group). **, *P* < .01 comparison of means (paired Student's *t* test).

To investigate whether the addition of a fast BK current could restore bursting in CORT-treated corticotrophs, cells were pretreated with 100 nM CORT for 150 minutes (155 ± 7 min; n = 6). Resting membrane potential of these CORT-treated cells was −65.0 ± 0.7 mV. Previously, costimulation with CRH and AVP was sufficient to induce activity in CORT-treated cells. However, stimulation with CRH alone induced a small depolarization but was unable to routinely reach threshold to induce an increase in event frequency, and therefore, a small leak current (*g_lk_*; typically 0.04 nS with *V_lk_*; 0 mV) was injected to induce activity. After CRH stimulation, predominantly single-spike activity was observed in CORT-treated cells (Figure 8A). In all cells (six of six), injection of a fast BK current with a dynamic clamp, during CRH stimulation, was sufficient to induce bursting in spiking cells or increase bursting in cells already displaying some bursting behavior (Figure 8B). Mean event duration was 53 ± 15 milliseconds and burstiness factor was 0.14 ± 0.07 in CORT-treated cells after CRH stimulation (Figure 8, C and D). Addition of a fast BK current resulted in a significant (*P* < .01) increase in both event duration (330 ± 61 msec) and burstiness factor (0.65 ± 0.10). In most cases, the parameters were as follows: *g_BK_* = +0.5 nS; *V_f_* = −10 mV; τ*_BK_* = 5 milliseconds; *g_lk_* = 0.04 pS.

## Discussion

These studies reveal that CORT, in the intermediate time domain of minutes to hours, is able to inhibit both spontaneous and CRH/AVP-evoked excitability through multiple effects on resting membrane potential, event frequency, and bursting activity. By using the dynamic clamp technique, we demonstrate experimentally that a fast activating BK current is an important component of bursting behavior that is primarily driven by CRH and suggest that control of BK channels represents a key mechanism for glucocorticoid-negative feedback. However, CORT can additionally regulate electrical excitability through a BK-independent mechanism, controlling primarily event frequency.

Previous studies using corticotrophs isolated from male and female mice (12, 13, 34) revealed a robust depolarization and increase in firing frequency accompanied by a characteristic transition from spiking to bursting after CRH/AVP stimulation in metabolically intact corticotrophs. Corticotrophs in vivo would typically be exposed to CRH and AVP after an acute stressor; however, our previous data revealed that CRH primarily promotes pseudoplateau bursting, whereas AVP increases single-spike frequency (13). Pseudoplateau bursting is characterized by oscillations of the membrane potential in a depolarized state, which increases intracellular Ca^2+^ to a greater extent than spiking and therefore is believed to be important in driving robust hormone secretion (15–17). BK channels have been shown to be an important component in the generation of bursting behavior in multiple anterior pituitary cell types (19, 35, 36), including corticotrophs (13). Because glucocorticoids have been reported to control BK channels through multiple mechanisms our aim was as follows: 1) to investigate whether BK channels are a target for glucocorticoid-negative feedback in corticotrophs and 2) to investigate whether glucocorticoids controlled electrical excitability of corticotrophs independently of BK channels. We thus analyzed the effects of CORT on both CRH/AVP-evoked excitability and interrogating the effect of CORT on pseudoplateau bursting evoked by CRH alone that is dependent on functional BK channels.

Glucocorticoid-negative feedback in the intermediate time domain (minutes to hours) in murine corticotrophs was associated with the control of both BK-dependent, and BK-independent, mechanisms controlling electrical excitability. In cells pretreated with CORT for 1–3 hours, the transition to bursting induced by CRH/AVP or CRH alone was significantly attenuated. Importantly, because CRH is the major driver to promote bursting, we demonstrated that subtraction of a fast BK channel conductance in CRH-stimulated corticotrophs, using a dynamic clamp, essentially reverted bursting back to single-spike activity. The lack of bursting in the absence of the fast BK conductance was similar to that observed in corticotrophs with a genetic deletion of total BK channels (BK^−/−^), in which CRH, as for CRH/AVP, could stimulate an increase in single-spike frequency. Importantly, in wild-type cells pretreated with 100 nM CORT, addition of a fast-activated BK conductance, in the presence of CRH, restored bursting activity similar to that observed using CRH in untreated wild-type corticotrophs. Taken together, the data support the hypothesis that a fast BK channel is a key regulator of transition to bursting activity and suggest that modulation of BK channel properties and/or regulation provide a likely mechanism for inhibition of corticotroph bursting by glucocorticoids. In the mathematical model, CRH-induced bursting is modeled by the activation of an L-type Ca^2+^ conductance along with a significant right shift in the activation curve of the BK channel population (BK-near) close to the Ca^2+^ channels and a decrease in the activation time constant. In the presence of CRH, the activation of these channels has to be significantly fast (<10 msec) to promote bursting; otherwise, the BK channel becomes inhibitory (13, 36).

How may glucocorticoids regulate BK-near to attenuate CRH-induced bursting? Glucocorticoids have been reported to control multiple aspects of BK channel properties and function in corticotrophs and other endocrine systems. These include nongenomic effects of glucocorticoids on BK channel activity mediated through accessory subunits (20, 21) as well as genomic mechanisms that control BK channel phosphorylation through control of protein phosphatase activity (22, 23) and switching of BK channel splice variant expression (24, 25). Some effects of CORT were observed after an approximately 10-minute exposure; however, the most robust inhibitory effects were observed after 1–2 hours. Although this suggests that genomic, rather than nongenomic, effects of CORT dominate, this remains to be determined. Changes in BK channel mRNA splicing may occur in the intermediate time scale; however, whether this would be reflected at functional changes in BK channel variant protein expression, or abundance at the plasma membrane, is unknown. However, an attractive working hypothesis is that glucocorticoid regulation is mediated through effects on BK channel phosphorylation. For example, protein kinase A (PKA)-dependent phosphorylation of the stress-regulated exon (STREX) variant of the BK channel is occluded by glucocorticoids in this time domain through the control of protein phosphatase activity (22, 23). Importantly, STREX is expressed in murine corticotrophs (12, 37), and PKA phosphorylation results in a significant right shift in the voltage activation curve of this variant, in contrast to all other known variants (38), as predicted by the model. In addition, the activation of the STREX variant would be expected to be sufficiently fast (<10 msec), under conditions of free Ca^2+^ experienced close to an open Ca^2+^ channel (23, 38–40), to promote bursting. Clearly, elucidation of the molecular components, and potential mechanism for glucocorticoid regulation, of the fast BK component remains a major challenge but warrants further study.

The above data reveal an important role for BK channels in control of bursting determined by the opposing actions of CRH and glucocorticoids. In addition, our data clearly demonstrate that CORT exerts multiple inhibitory effects on corticotroph electrical excitability, in part dependent on the duration of CORT exposure. For example, CORT both inhibits the CRH-dependent transition to bursting and reduces the spike frequency. CRH acts primarily to induce bursting, whereas AVP predominantly increases event frequency (13). This suggests that the distinct BK-independent and BK-dependent ionic mechanisms underlying both spiking and bursting, respectively, can be regulated by CORT. Moreover, in corticotrophs in which BK channels have been genetically deleted, both CRH- and CRH/AVP-induced stimulation of single-spike frequency was attenuated by CORT in the intermediate time domain. Although longer exposure to CORT may delay the onset of secretagogue-induced excitability, CORT did not significantly affect the amplitude of the initial CRH/AVP-induced depolarization toward the threshold required for increased action potential frequency. This suggests that CORT does not, in general, prevent the ionic mechanisms leading to initial depolarization by CRH and/or AVP. In addition, a significant hyperpolarization was observed in CORT_90_ cells compared with controls, which could partially explain a reduction in spontaneous event frequency. However, this hyperpolarization was not observed in CORT_150_ cells, which showed a further reduction in both spontaneous and CRH/AVP-evoked activity. These data reveal that CORT is also able to regulate spontaneous and CRH/AVP activity via multiple ionic mechanisms through temporally distinct effects on resting membrane potential or spiking/bursting.

In corticotrophs, based on both experimental data and modeling analysis, several different conductances have been proposed to control resting membrane potential and the initial depolarization in response to CRH/AVP. Resting membrane potential is typically −55 mV, and previous studies have shown that a background sodium conductance is important in setting the membrane potential of corticotrophs and other pituitary cells; replacement of external Na^+^ with the large organic cation N-methyl-D-glucamine results in a membrane hyperpolarization and cessation of spontaneous activity (12, 41). Mathematical modeling predicts that the nonselective Na^+^ conductance is a major determinant of membrane potential and a key target for CORT. A decrease in spontaneous activity was simulated by a reduction in background Na^+^ conductance, which causes a slight hyperpolarization and reduces the likelihood of reaching threshold, thus decreasing frequency. Although the molecular identity of this background Na^+^ conductance is yet to be identified, work in other pituitary cell types suggests that transient receptor potential-like conductances may play a role and have been shown to be regulated by the cAMP/PKA pathway (41). Alternatively, CORT could regulate membrane potential through other K^+^ conductances. For example, a Tandem of P-domains in a Weakly Inward rectifying K^+^ channelrelated 1 like conductance has been reported to control resting membrane potential and be inhibited by supraphysiological levels of CRH and AVP in murine corticotrophs to promote membrane depolarization (42, 43). However, whether these conductances, or others that may be involved in resting membrane potential and/or CRH/AVP induced depolarization, such as T-type (44) and P/Q-type Ca^2+^ channels (45) or inwardly rectifying K^+^ channels (46), are controlled by CORT is unknown.

In conclusion, we have demonstrated that glucocorticoids can modulate electrical excitability of corticotroph cells through multiple mechanisms. CORT controls bursting through control of BK-dependent mechanisms, whereas CORT effects on spike frequency are mediated via BK-independent mechanisms including control of conductances important for control of membrane potential. The ability of CORT to regulate multiple ionic mechanisms that control corticotroph excitability is likely of physiological importance due to the diverse and powerful effects of glucocorticoids to control ACTH secretion. For example, the ability to engage distinct mechanisms may be important for the corticotroph to modulate their output in the face of the oscillations of plasma glucocorticoids throughout the day due to both circadian and ultradian rhythms (47, 48) as well in response to acute and chronic stress. The HPA axis function is dependent on effective glucocorticoid-negative feedback, which is often impaired in patients suffering from depression and anxiety disorders (2). Further understanding of glucocorticoid-negative feedback at the corticotroph level could provide novel insight into the treatment of stress and stress-related disorders.
